# Methylated *Vnn1* at promoter regions induces asthma occurrence via the PI3K/Akt/NFκB-mediated inflammation in IUGR mice

**DOI:** 10.1242/bio.049106

**Published:** 2020-04-28

**Authors:** Yan Xing, Hongling Wei, Xiumei Xiao, Zekun Chen, Hui Liu, Xiaomei Tong, Wei Zhou

**Affiliations:** 1 Department of Pediatrics, Peking University Third Hospital, Beijing 100191, China; 2 Department of Laboratory Medicine, Peking University Third Hospital, Beijing 100191, China; 3 Department of Social Medicine and Health Education, School of Public Health, Peking University, Beijing 100191, China

**Keywords:** Vascular non-inflammatory molecule 1 (vannin-1), Methylation, Protein kinase B (Akt), NFκB, Asthma, Intrauterine growth retardation (IUGR)

## Abstract

Infants with intrauterine growth retardation (IUGR) have a high risk of developing bronchial asthma in childhood, but the underlying mechanisms remain unclear. This study aimed to disclose the role of vascular non-inflammatory molecule 1 (vannin-1, encoded by the *Vnn1* gene) and its downstream signaling in IUGR asthmatic mice induced by ovalbumin. Significant histological alterations and an increase of vannin-1 expression were revealed in IUGR asthmatic mice, accompanied by elevated methylation of Vnn1 promoter regions. In IUGR asthmatic mice, we also found (i) a direct binding of HNF4α and PGC1α to Vnn1 promoter by ChIP assay; (ii) a direct interaction of HNF4α with PGC1α; (iii) upregulation of phospho-PI3K p85/p55 and phospho-Akt^Ser473^ and downregulation of phospho-PTENT^yr366^, and (iv) an increase in nuclear NFκB p65 and a decrease in cytosolic IκB-α. In primary cultured bronchial epithelial cells derived from the IUGR asthmatic mice, knockdown of Vnn1 prevented upregulation of phospho-Akt^Ser473^ and an increase of reactive oxygen species (ROS) and TGF-β production. Taken together, we demonstrate that elevated vannin-1 activates the PI3K/Akt/NFκB signaling pathway, leading to ROS and inflammation reactions responsible for asthma occurrence in IUGR individuals. We also disclose that interaction of PGC1α and HNF4α promotes methylation of Vnn1 promoter regions and then upregulates vannin-1 expression.

## Abstract (precis)

**Summary:** Vascular non-inflammatory molecule 1 (vannin-1) is elevated in intrauterine growth retardation (IUGR) asthmatic mice, leading to activation of the PI3K/Akt/NFκB pathway responsible for bronchial oxidative stress and hyperresponsiveness.

## INTRODUCTION

In recent years, the survival rate of premature and low birth weight infants has increased year by year globally with the continuous development of perinatal medicine, assisted reproductive technology and rescue technology for infants. A significant proportion of these low birth weight infants have intrauterine growth retardation (IUGR), which is defined as a fetal birth weight below the tenth percentile of their gestational age (Sharma et al., 2016a,b). Epidemiological surveys have shown a significant increase in the risk of developing bronchial asthma in childhood and adulthood in IUGR children (Gatford et al., 2017).

With the increasing incidence of IUGR, the pathogenesis and physiological mechanism of bronchial asthma have been continuously recognized and updated. At present, it mainly focuses on inflammation, immune response changes and airway remodeling caused by abnormal subepithelial myofibroblasts and chronic inflammation. Studies have shown that abnormalities in multiple signaling pathways in lung tissue involve the development and progression of asthma, such as PI3K/Akt, MAPK, ERK, JAK and c-Jun (El-Hashim et al., 2017; Li et al., 2015; Southworth et al., 2018; Wagh et al., 2017; Yang et al., 2018). In particular, many studies have shown that activation of the PI3K/Akt pathway plays an important role in the development of asthma by activating oxidative stress and inflammatory responses (Wagh et al., 2017; Yang et al., 2018). The serine/threonine kinase Akt, also known as protein kinase B (PKB), is activated by lipid products of phosphatidylinositol 3-kinase (PI3K). However, the molecular mechanism of bronchial asthma in IUGR children is not clear.

Recent studies have shown that DNA methylation abnormalities may be associated with a predisposition to obesity, insulin resistance and diabetes, and hypertension in IUGR adulthood. It was reported that DNA methylation of exon 2 of dual specificity phosphatase 5 (DUSP5) in IUGR rats caused an increase in mRNA expression, which led to insulin resistance by regulating Ras-MAPK signaling pathway activity (Fu et al., 2006). Increased phosphorylation of the insulin receptor substrate (IRS) leads to the development of insulin resistance. In addition, postnatal overnutrition in IUGR rats can upregulate DNA methylation levels at specific sites of peroxisome proliferator activated receptor γ coactivator-1α (PGC1α), promoting the development of insulin resistance, in which PI3K/Akt activity is reduced (Xie et al., 2015). Vascular non-inflammatory molecule 1 (vannin-1) is a GPI-anchored cell surface protein encoded by the *Vnn1* gene. Vannin-1 is a newly discovered molecule and possesses pantetheinase activity, which plays a role in inflammation regulation and oxidative-stress response. Human and mouse *Vnn1* have a high homology of 80%. In childhood asthma, it has been found that increased mRNA levels in the *Vnn1* gene were associated with hormone sensitivity (Xiao et al., 2015). Therefore, this study aimed to investigate the regulatory role of *Vnn1* on the PI3K/Akt signaling activity in the IUGR mice challenged with ovalbumin (OVA) in order to discover the potential molecular mechanisms of asthma in IUGR children.

## RESULTS

### Asthma is induced in the nmIUG and the IUGR mice

**Fig. 1.**
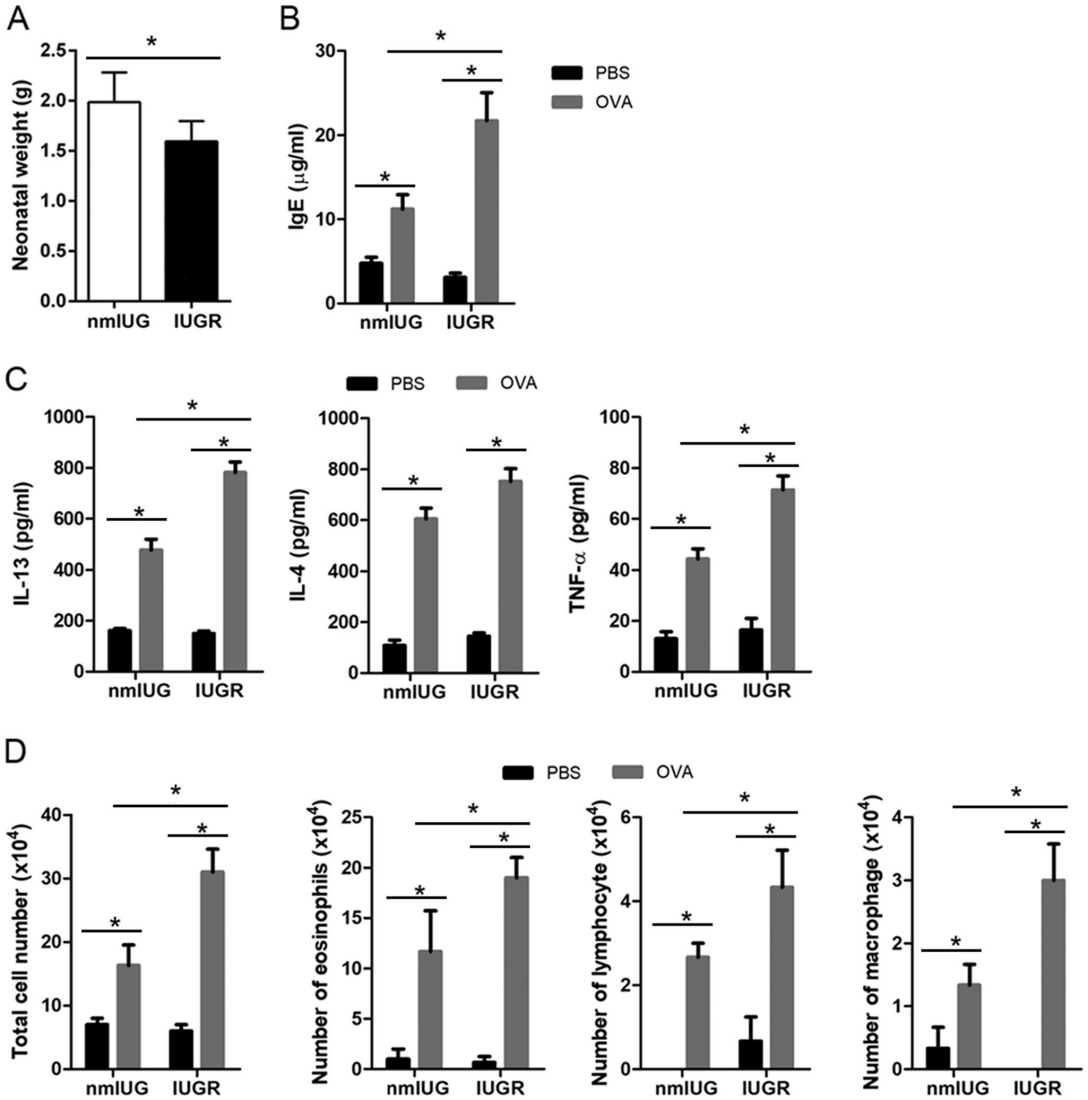
**Establishment of asthma in IUGR mice.** IUGR was established by feeding pregnant mice with a low protein diet. (A) 6 h after birth weight was measured, which showed a significant reduction in the IUGR group in comparison with the normal intrauterine growth (nmIUG) group. Asthma was induced with OVA in the IUGR and the nmIUG groups. PBS induction was used as the control. (B) The concentration of IgE in serum was measured using ELISA kit. (C) Bronchi alveolar lavage fluid (BALF) was collected, and the levels of IL-13, IL-4 and TNF-α were assessed with ELISA assays. (D) The number of eosinophils, lymphocytes and macrophages in BALF was counted and compared. Data are shown as mean±s.d. *n*=16 (A) and 8 (B–D). *P<0.01.

As previously described (Fu et al., 2006; Xing et al., 2019), the normal intrauterine growth (nmIUG) and the IUGR pups were produced by feeding female mice with normal and low protein diets, respectively. Birth weight was measured at 6 h, showing a significant (P<0.01) reduction in the IUGR group (1.15±0.24 g) compared to the nmIUG group (1.85±0.52 g) (Fig. 1A).

Asthma was then induced with OVA in 6-week-old IUGR and nmIUG mice, whereas the PBS inductions were used as the controls. Asthma is a chronic inflammatory airway disease in which interleukin-4 (IL-4), IL-13, and TNF-α are involved (Manni et al., 2016). These inflammatory factors promote airway eosinophilia infiltration, mucus overproduction, bronchial hyperresponsiveness and immunoglobulin E (IgE) synthesis (Manni et al., 2016). The IgE levels were measured in the serum, showing a dramatic elevation (*P*<0.01) in the OVA group compared to the PBS controls, both in the nmIUG and IUGR groups (Fig. 1B). To evaluate the inflammation reactions of bronchi, bronchoalveolar lavage fluid (BALF) was collected. Compared to the PBS controls, the levels of IL-13, IL-4 and TNF-α were increased significantly (*P*<0.01) both in the nmIUG and the IUGR mice challenged with OVA (Fig. 1C). Cells were also classified and counted in the BALF. Compared to the PBS controls, the number of eosinophils, lymphocytes and macrophages as well as the total cell numbers were significantly higher (P<0.01) both in the nmIUG-OVA and the IUGR-OVA groups (Fig. 1D). Notably, the levels of IgE, IL-13 and TNF-α as well as the numbers of the inflammatory cells in the BALF were significantly higher (*P*<0.01) in the IUGR-OVA group than in the nmIUG-OVA group (Fig. 1B–D).

**Fig. 2.**
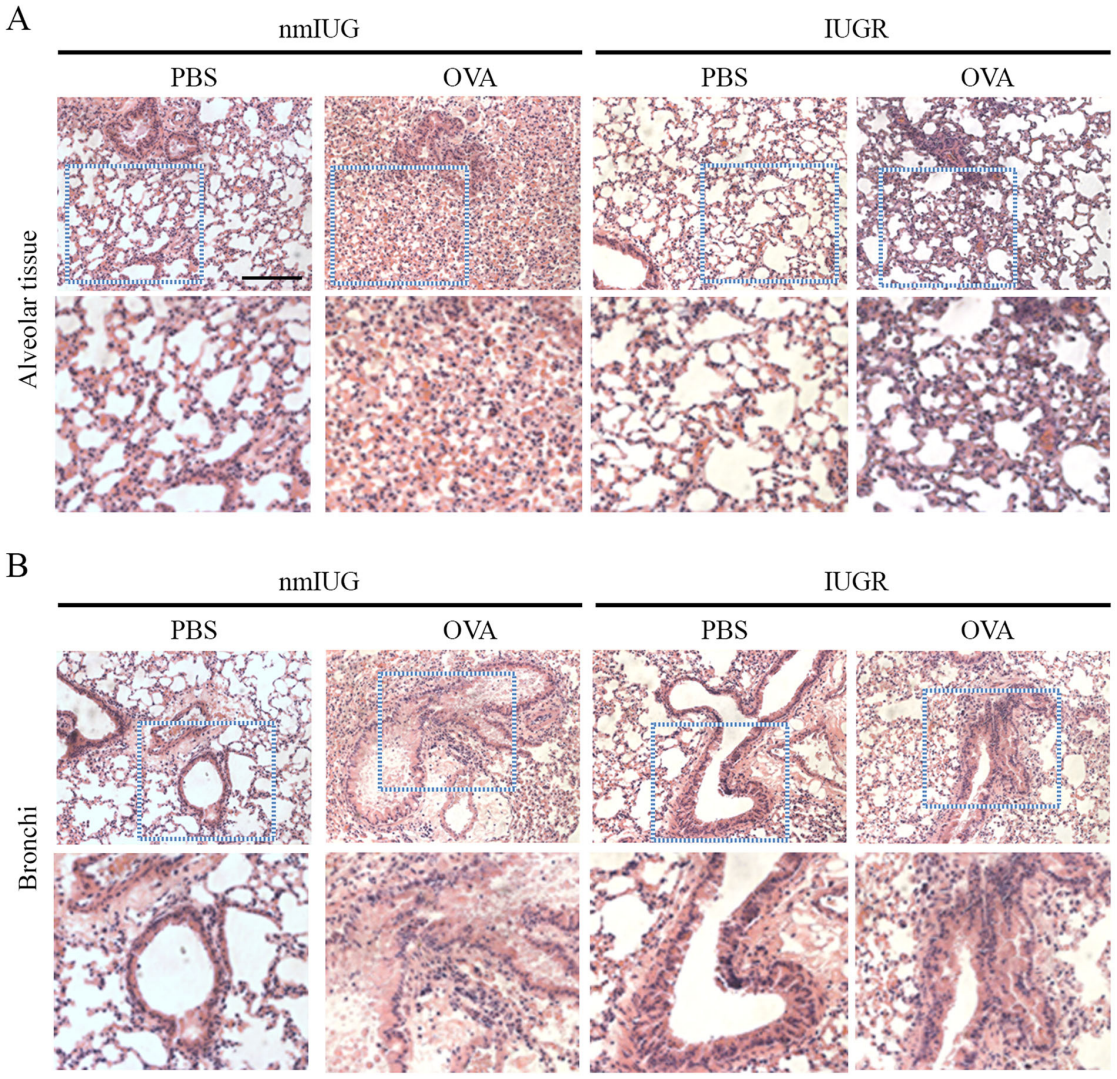
**Lung tissue was stained with H&E** **in the asthmatic mice.** Asthma was induced with OVA in the IUGR and the nmIUG mice. PBS induction was used as the control. Lung tissue was prepared for H&E staining. Representative pictures of alveolar (A) and bronchi (B) tissue are shown. Higher-magnification images indicated by dashed boxes are provided in the bottom panels. Original magnification ×400. Scale bar: 50 µm.

**Fig. 3.**
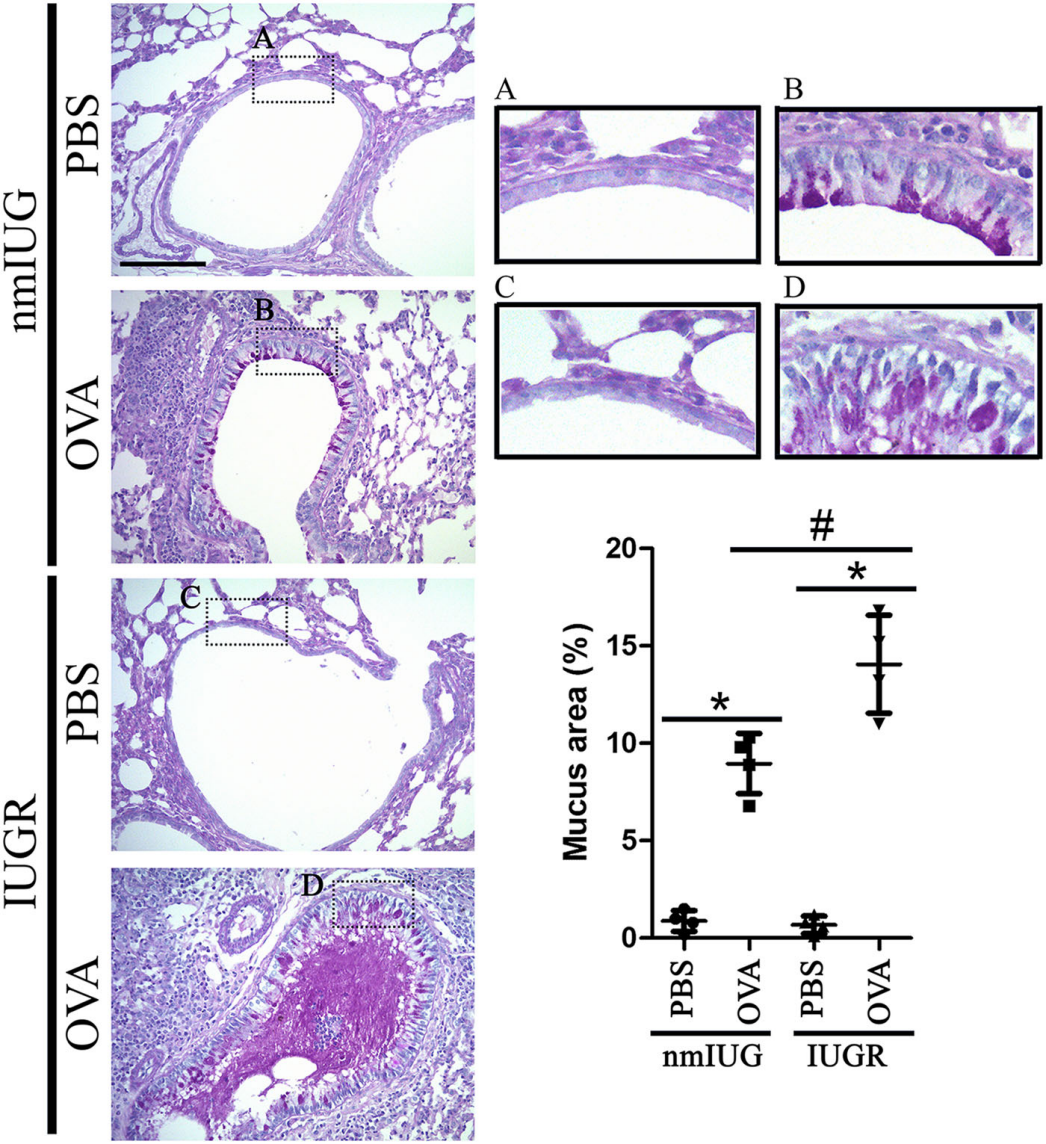
**Lung tissue was stained with periodic acid-Schiff (PAS) in IUGR and nmIUG asthmatic mice.** Asthma was induced with OVA in IUGR and nmIUG mice. PBS induction was used as the control. Lung tissue was prepared for PAS staining to reveal mucus production. Representative images and higher-magnification images indicated by dashed boxes are provided. The surface area of mucin-containing goblet cells per total surface area of airway epithelial basal membrane was quantitated and compared. Original magnification ×400. Scale bar: 50 µm. Data are shown as mean±s.d. *n*=4. *P<0.001 versus PBS, #*P*<0.05 IUGR-OVA versus nmIUG-OVA.

Hematoxylin and Eosin (H&E) staining was performed on lung tissue. We observed obvious eosinophil infiltration in alveolar tissue (Fig. 2A) and bronchi (Fig. 2B) in the OVA challenged mice, particularly in the IUGR mice. We also performed periodic acid-Schiff (PAS) staining, which showed that the surface area of mucin-containing goblet cells was markedly increased in OVA-induced asthmatic mice compared to that in PBS controls (Fig. 3). Moreover, IUGR-OVA mice had more serious mucus production in the bronchial airway than nmIUG-OVA mice (Fig. 3). These findings demonstrated that the asthma model had been successfully induced, and more severe asthmatic inflammation was observed in the bronchi of the IUGR mice.

### Expressions of *Vnn1* are elevated in asthmatic IUGR mice

**Fig. 4.**
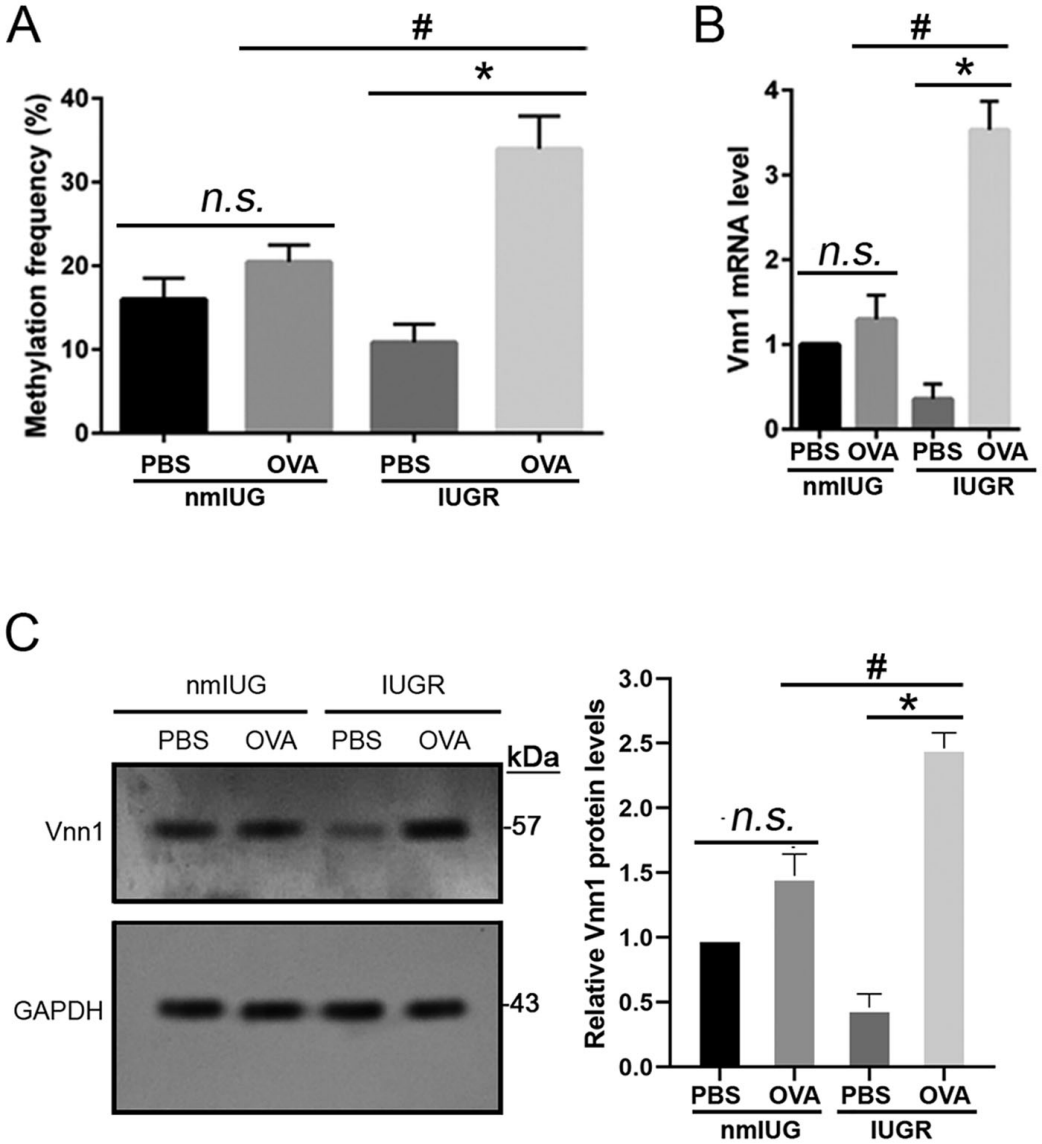
**The methylation and expression levels of *Vnn1* in IUGR and nmIUG asthmatic mice.** Asthma was induced with OVA in IUGR and nmIUG mice. PBS induction was used as the control. (A) Total DNA was extracted and sequencing of the CpG islands in *Vnn1* promoter regions was performed to assess the methylation levels of *Vnn1* promoter. (B) Total RNA was extracted from lung tissues, qPCR was used for assessing expressions of *Vnn1* at the mRNA level. (C) Total protein was extracted from lung tissues, and immunoblot assay was performed for expressions of *Vnn1* at the protein level. Data are shown as mean±s.d. *n*=8. *P<0.001, #*P*<0.05.

It has been reported that the methylation status of *Vnn1* has obvious impacts on its mRNA level (Xiao et al., 2015). In this study, we first assessed the methylation levels of *Vnn1* at the promoter regions in the asthmatic IUGR and nmIUG mice. Our data showed that compared to the PBS controls, the methylation frequency of CpG islands of *Vnn1* at promoter regions was significantly elevated (*P*<0.01) in the IUGR-OVA group, but not the nmIUG-OVA group (Fig. 4A). Consistent with this finding and in comparison with the PBS controls, we detected a significant (*P*<0.001) increase of vannin-1 expression both at the mRNA and protein level in the IUGR-OVA group, but not in the nmIUG-OVA group (Fig. 4B,C). Therefore, the function of vannin-1 was investigated in the asthmatic IUGR mice in the following experiments.

### PI3K/Akt signaling is activated in asthmatic IUGR mice

**Fig. 5.**
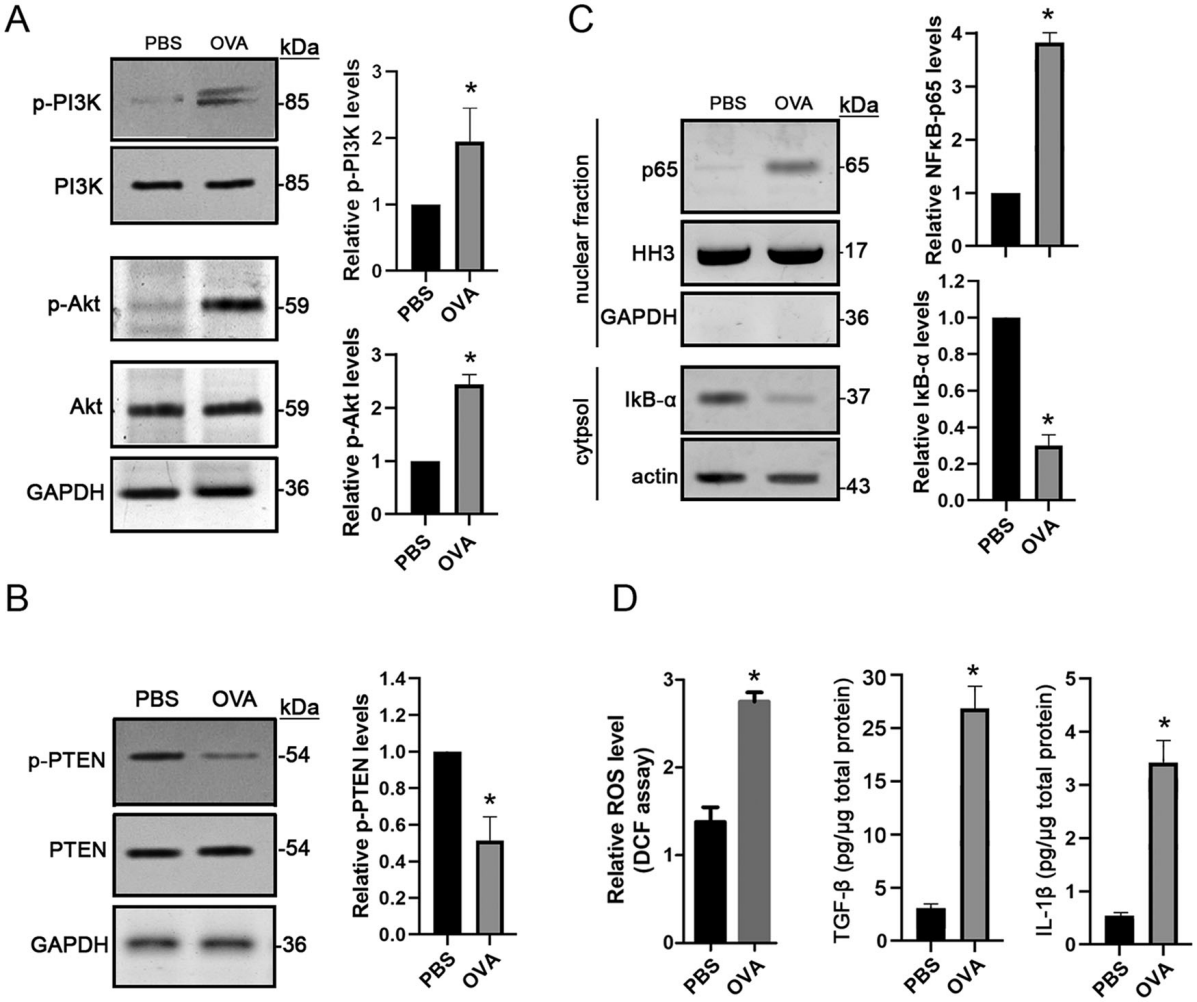
**Activation of the PI3K/Akt and NFκB signaling in IUGR asthmatic mice.** Asthma was induced with OVA in IUGR mice. PBS induction was used as the control. Total protein as well as the cytosolic and nuclear protein were extracted from lung tissues. (A–C) Immunoblot assay was performed for expressions of phospho-PI3K/p85Tyr458/p55Tyr199 and phospho-AktSer473 (A), and phospho-PTENTyr336 (B) as well as cytosolic IκB-α and nuclear NEκB-p65 subunit (C). (D) The levels of ROS, IL-1β and TGF-β in lung tissues were assessed using DCF assay and ELISA, respectively. Data are shown as mean±s.d. *n*=8. *P<0.001 OVA versus PBS.

The PI3K/Akt signaling pathway plays an important role in the release of various cytokines and inflammatory factors (Martini et al., 2014). In the IUGR asthmatic mice, we evaluated its activation levels in the lysates isolated from lung tissues. Immunoblot assays showed that the phospho-PI3K p85^Tyr458^/p55^Tyr199^ and phospho-Akt^Ser473^ levels were significantly increased (*P*<0.001) in the OVA group compared to the PBS controls (Fig. 5A). PTEN is a critical negative regulation kinase for PI3K/Akt activation (Martini et al., 2014). In this study, reduction of the phospho-PTEN^Tyr366^ was detected in the OVA group (Fig. 5B). Previous studies suggest that Akt regulates transcriptional activity of nuclear factor-κB (NFκB) by inducing phosphorylation and subsequent degradation of inhibitor of κB (IκB). NFκB, a family of transcription factors, regulates diverse cellular activities related to inflammation and immune responses (Chauhan et al., 2018). Therefore, we assessed the nuclear levels of NFκB. Our findings show that the nuclear NFκB abundance was increased dramatically (*P*<0.001) in the OVA group, which was accompanied by a reduction of IκB-α in the cytosolic fractions (Fig. 5C). In the lung tissue lysates, the levels of reactive oxygen species (ROS), TGF-β and IL-1β were also significantly elevated (*P*<0.001) in the OVA group compared to the PBS controls (Fig. 5D). These findings suggest that the PI3K/Akt signaling pathway plays a critical role in the development of asthma at least partially by NFκB-mediated production of ROS, IL-1β and TGF-β.

### Association of PGC1α and HNF4α with *Vnn1* in asthmatic IUGR mice

**Fig. 6.**
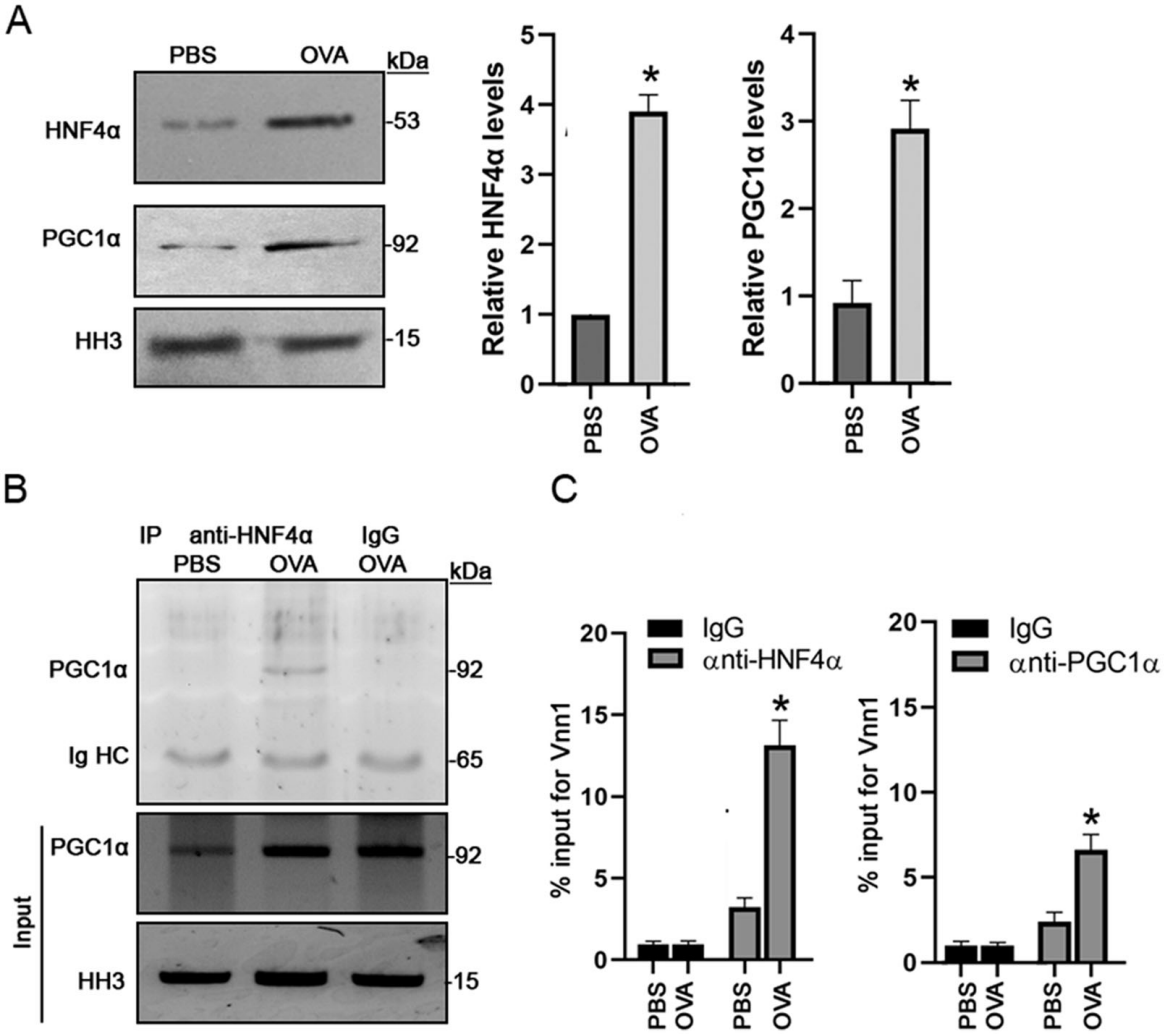
**PGC1α and HNF4α interacts and binds to *Vnn1* promoter in IUGR asthmatic mice.** Asthma was induced with OVA in IUGR mice. PBS induction was used as the control. Nuclear protein was extracted from lung tissues. (A) Immunoblot assay was performed for expressions of PGC1α and HNF4α. (B) IP was performed using mouse anti-HNF4α antibody and Protein G-coupled agarose beads, followed by immunoblot with rabbit-anti-PGC1α antibody. The normal mouse IgG was used as the IP control. (C) In primary cultured bronchial epithelia cells isolated from IUGR mice injected with OVA or PBS, ChIP assay was performed using mouse anti-HNF4α or rabbit anti-PGC1α antibodies. The normal mouse or rabbit IgG was used as the control. Graphics show the percentage of total DNA immunoprecipitated by each indicated antibody. Data are shown as mean±s.d. *n*=4. *P<0.01 OVA versus PBS.

As described above, the methylation frequency of *Vnn1* promoter region was elevated in the IUGR mice following asthma induction. It was reported that PGC1α is a key upstream regulator for *Vnn1* transcription in liver gluconeogenesis, in which hepatocyte nuclear factor-4α (HNF4α) is required (Chen et al., 2014). Therefore, we assessed the levels of PGC1α and HNF4α in the nuclear fractions from lung tissue. Our results show that the abundance of both PGC1α and HNF4α was significantly increased (*P*<0.001) in the OVA group compared to the PBS controls (Fig. 6A). Further evaluation using immunoprecipitation assay revealed a remarkable interaction between PGC1α and HNF4α in the OVA group (Fig. 6B). To test if PGC1α and HNF4α regulated *Vnn1* transcription levels through binding to its promoter regions, we performed a ChIP assay with anti-PGC1α and anti-HNF4α antibodies followed by qPCR using specific primers for *Vnn1* promoter. The binding ability of PGC1α and HNF4α to the *Vnn1* promoter was calculated as a percentage of DNA precipitated relative to the total input. We found that both PGC1α and HNF4α – in particular HNF4α – bound to a greater extent to the *Vnn1* promoter in the OVA group compared to the PBS controls (Fig. 6C).

### Knockdown of *Vnn1* inhibits Akt activation as well as inflammatory cytokines and ROS production in primary bronchial epithelial cells isolated from asthmatic IUGR mice

**Fig. 7.**
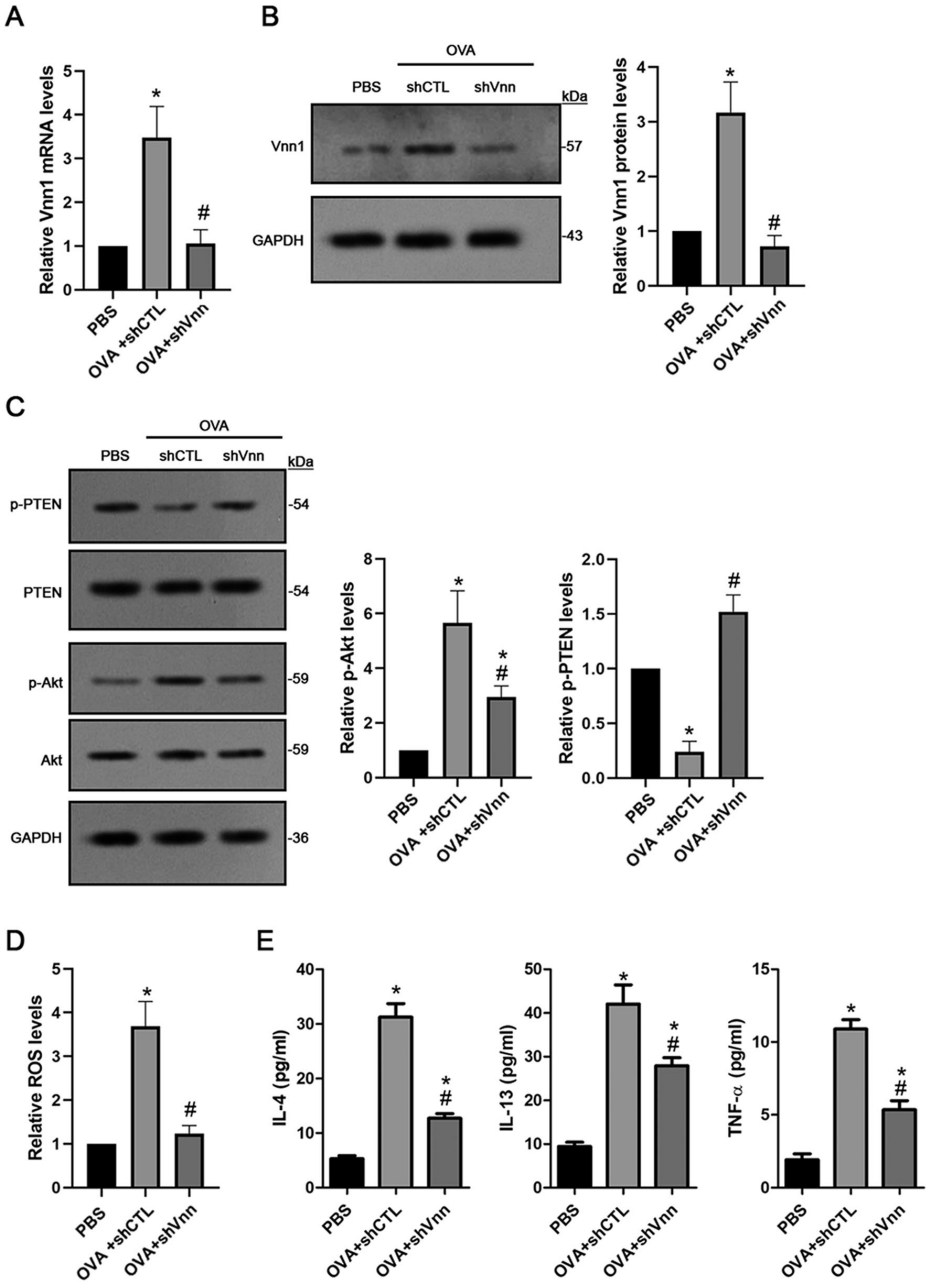
***Vnn1* knockdown inhibits Akt activation and ROS production in primary cultured bronchial epithelial cells derived from IUGR asthmatic mice.** Knockdown of *Vnn1* was performed using shVnn specifically targeted against mouse *Vnn1* gene in primary cultured bronchial epithelial cells isolated from IUGR mice injected with OVA or PBS. The control shRNA (shCTL) did not target against any mouse genes. (A) The mRNA level of *Vnn1* was quantitatively assessed using qPCR. (B) The protein level of *Vnn1* was measured using an immunoblot assay. (C) The protein level of phospho-PTENTyr366 and phospho-AktSer473 was assessed using immunoblot assay. (D) The ROS level was assessed using DCF assay. (E) The levels of IL-4, IL-13 and TNF-α in the supernatant were measured using ELISA. Data are shown as mean±s.d. *n*=3. *P<0.001 OVA +shCTL versus PBS, #*P*<0.01 OVA+shVnn versus OVA+shCTL.

To verify if vannin-1 directly regulated the PI3K/Akt signal required for asthma occurrence in IUGR mice, we knocked down *Vnn1* expression using lentiviral shRNA specifically targeted against mouse *Vnn1* and evaluated activation levels of the phospho-Akt^Ser473^ in primary cultured bronchial epithelia cells. The mRNA and protein levels of *Vnn1* were dramatically increased (*P*<0.001) in cultured cells from the IUGR asthma mice, which was significantly (*P*<0.05) prevented by shRNA-Vnn1 (Fig. 7A,B). Reduction of phospho-PTEN^Tyr366^ and increase of phospho-Akt^Ser473^ activity was dramatically (*P*<0.01) inhibited by shRNA-Vnn1 in primary bronchial epithelia cells isolated from IUGR asthmatic mice (Fig. 7C). In addition, elevation of ROS production was also prevented (*P*<0.01) by shRNA-Vnn1 in the cells isolated from IUGR asthmatic mice (Fig. 6D). In addition, we assessed the levels of inflammatory cytokines in the cultured media. A significant increase in IL-13, IL-4 and TNF-α (*P*<0.001) was detected in the OVA group compared to the PBS controls, which was dramatically (*P*<0.01) prevented by shRNA-Vnn1.

## DISCUSSION

An asthma model was successfully induced in the current study (Figs 1–3). We found that PI3K and Akt activity was increased significantly in IUGR asthmatic mice (Fig. 5A). Similarly, it has been reported that expression of PI3K was elevated in a rat asthma model (Xia et al., 2012). The use of the PI3K inhibitor alleviated airway inflammation and hyperresponsiveness through reduction of nitric oxide, which is closely related to the development of asthma (Xia et al., 2012). In a mouse asthma model, it was also found that upregulation of the phospho-Akt was involved the occurrence of asthma (Cheng et al., 2011). Therefore, these findings suggest that PI3K/Akt signaling plays a critical role in the pathogenesis of asthma in IUGR mice.

Vannin-1, which is encoded by the gene *Vnn1*, is an epithelial ectoenzyme with pantetheinase activity that provides cysteamine/cystamine to tissues and is implicated in redox homeostasis (Berruyer et al., 2004). The expression level of *Vnn1* in lungs was not altered in experimental mouse asthma models challenged by repeated allergen or IL-13 (Lewis et al., 2009; Zimmermann et al., 2004). Asthma developed in *Vnn1*-knockout mice following a challenge by house dust mites (Xiao et al., 2015). Interestingly, it was reported that the asthma patients with downregulation of the *Vnn1* gene expression level were not sensitive to glucocorticoid therapy. Absence of the *Vnn1* gene resulted in resistance to dexamethasone treatment, which was reflected by persistent airway hyperresponsiveness and inflammatory cells in the lungs in an asthma mouse model (Xiao et al., 2015). These findings suggest that vannin-1 may contribute to optimal host response to corticosteroid treatment. Nevertheless, in an experimental mice model, *Vnn1*-knockout mice exhibited resistance to oxidative injury induced by whole-body irradiation, presenting with a reduction of inflammatory responses to ROS inducers in the thymus (Berruyer et al., 2004), suggesting that vannin-1 also involved the production of ROS and oxidative stress reaction. Moreover, we found that expression of vannin-1 in lung tissue significantly increased both at mRNA and protein levels in IUGR asthmatic mice, but not in nmIUG mice (Fig. 4B,C). These findings imply that upregulation of vannin-1 did occur in IUGR mice with asthma, and that asthma in IUGR mice that have increased vannin-1 may respond better to corticoid treatment. To investigate how *Vnn1* expression levels are regulated, we analyzed the methylation frequency of *Vnn1* at promoter regions. Our results showed significant upregulation of methylation of *Vnn1* promoter in the IUGR asthmatic mice, but not in the nmIUG mice (Fig. 4A). Methylation of CpG sites at promoter regions is generally thought to cause gene silencing (Hon et al., 2012). However, positive correlations between promoter methylation and increased gene expression have been reported (Wagner et al., 2014). It was found that DNA methylation changes in nitric oxide signaling systems such as nitric oxide synthase and arginase are associated with chronic cardiopulmonary disease in adults with IUGR (Xie et al., 2015). It should be noted that a more serious degree of asthma was developed in the IUGR mice, presenting with more significant eosinophil infiltration, mucus accumulation and inflammatory cytokines production (Figs 1–3). In addition, methylation of both the *Vnn1* promoter region and its expression level increased dramatically only in the IUGR asthmatic mice (Fig. 4). Thus, elevation of vannin-1 may be responsible for the prominent histological alterations of lung tissues in the IUGR asthmatic mice. Nevertheless, the precise mechanism by which vannin-1 is upregulated in IUGR mice following OVA challenge should be investigated further.

In this study, we explored how transcription of *Vnn1* gene is regulated in the IUGR asthmatic mice. Vannin-1 is a liver-enriched oxidative stress sensor that has been implicated in the regulation of multiple metabolic pathways. It has been found that the *Vnn1* promoter has two HNF4α binding sites and that HNF4α can mediate the activation of *Vnn1* transcription by recruiting PGC1α, which plays a crucial role in the regulation of gluconeogenesis (Chen et al., 2014). Thus, we assessed expression levels of HNF4α and PGC1α in our model. Our data show that the protein levels of both HNF4α and PGC1α were significantly elevated in lung tissues (Fig. 6A), and an interaction between HNF4α and PGC1α was also detected in the IUGR asthmatic mice (Fig. 6B). Furthermore, ChIP assay shows that PGC1α and HNF4α, and in particular HNF4α, bound to a great extent to the *Vnn1* promoter (Fig. 6C). In a diabetic model, increased *Vnn1* induced a reduction in Akt phosphorylation, which might be associated with insulin resistance (Chen et al., 2014). However, we detected an elevation of PI3K/Akt activity in the IUGR asthmatic mice (Fig. 5A), which was supported by a reduction in phospho-PTEN^Tyr366^, a negative regulator of PI3K/Akt signaling (Fig. 5B). Akt is activated by the lipid products of PI3K and phosphorylates a variety of protein targets such as IκB, a key regulator of NFκB pathway that controls cell survival, proliferation and motility (Chauhan et al., 2018; Martini et al., 2014). Upon stimulation, IκB is phosphorylated at critical serine residues, resulting in polyubiquitination and degradation. In our study, we found a significant increase of NFκB p65 subunit in the nuclei and a decrease of IκB-α in the cytosolic fractions (Fig. 5C), suggesting that the NFκB pathway was activated and involved in asthma in IUGR mice.

To further demonstrate if *Vnn1* induced the PI3K/Akt pathway activation, we isolated and performed a primary culture of bronchial epithelial cells isolated from the IUGR asthmatic mice lungs. Thereafter, we did a knockdown assay by infecting the cells with validated shRNA specifically targeted against mouse *Vnn1* gene. Our data show that *Vnn1* expression was dramatically reduced by shRNA-Vnn1 (Fig. 7A,B). *Vnn1* knockdown also decreased the level of phospho-Akt^Ser473^ and increased phosphor-PTEN^Tyr366^ (Fig. 7C). Moreover, increased ROS and inflammatory cytokines such as IL-4, IL-13 and TNF-a production were suppressed by shRNA-Vnn1 in the cells from IUGR asthmatic mice (Fig. 7D,E). These findings may indicate a direct role of *Vnn1* in the development of allergic airway inflammation in IUGR asthmatic mice.

Taken together, our findings demonstrate that following OVA challenge, interaction of HNF4α and PGC1α increased methylation frequency of *Vnn1* at promoter regions and thus upregulated its expression, resulting in activation of the PI3K/Akt/NFκB signaling being responsible for ROS production and inflammatory mediators release, and finally resulting in asthma occurrence in the IUGR mice. We may provide a potential therapeutic target in asthma in IUGR children.

## MATERIALS AND METHODS

### Animals and experimental design

This study was carried out in strict accordance with the recommendations in the Guide for the Care and Use of Laboratory Animals of the Peking University. The protocol was approved by the Committee on the Ethics of Animal Experiments of the Peking University Third Hospital (protocol number: LA2017200). IUGR mice model was established as described previously (Fu et al., 2006; Xing et al., 2019). BALB/c mice were purchased from Laboratory Animal Science Department of Peking University Health and Science Center (Beijing, China).

20 female mice were mated with males overnight and pregnancy was verified by examining the vaginal sperm plugs. Pregnant mice were randomly fed an isocaloric (30.50 Kcal/g) diet containing 8% protein (low-protein diet) or 20% protein diet (normal diet) from day 1 of pregnancy until the birth of their pups. Both diets were obtained from Beijing Huakang Biotechnology Co., Ltd. (Beijing, China). Weights were recorded at birth, and IUGR pups were defined as and confirmed by having a lower birth weight minus 2 standard deviations than the controls. In this study, male pups were studied in order to avoid gender and hormonal influence. All pregnant mice during lactation and newborn rats after weaning at 21 days of age, were fed the normal diet. Fresh diet and water were provided daily *ad libitum*. An animal technician who was not involved in the assessments’ outcome performed the diet assignment.

At 6 weeks after birth, asthma was induced in the nmIUG and the IUGR pups. For the asthma group, mice were sensitized with two intraperitoneal injections of 100 mg ovalbumin (OVA; cat. no. vac-pova; InvivoGen, San Diego, CA, USA) emulsified in aluminum hydroxide (cat. no. vac-alu-250; InvivoGen) with a 2-week interval. The animals were then challenged with daily inhalation of OVA for 2 weeks. For the control group, the mice were injected intraperitoneally and inhaled with normal saline. Anesthesia was performed using isoflurane (2% inhalant), and all efforts were made to minimize suffering.

Blood was collected from the angular vein, and plasma was separated, frozen in dry ice and kept at −80°C until analysis. BALF was collected for inflammatory cell counts. Mice were then euthanized by cervical dislocation under anesthesia. The lung tissue was dissected. One part was prepared for H&E and periodic acid-Schiff (PAS) staining. The other parts were weighed, snap frozen in liquid nitrogen and stored at −80°C for further analysis.

### ELISA assay

The levels of serum IgE as well as tissue IL-1β and TGF-β were measured using mouse IgE ELISA kit (cat. no. ab157718; Abcam, Cambridge, MA, USA), mouse IL-1β ELISA kit (cat. RAB0275; Millipore/Sigma, Burlington, MA, USA), and mouse TGF-β1 ELISA kit (cat. LS-F5184-1; LifeSpan BioSciences, Seattle, WA, USA), respectively. The level of IL-13, IL-4 and TNF-α in BALF and *ex vivo* cultured cell media were measured using mouse IL-4 Quantikine ELISA Kit (cat. no. SM4000B1; R&D Systems, Minneapolis, MN, USA), mouse IL-13 ELISA Kit (cat. no. BMS6015; Invitrogen/Thermo Fisher Scientific, Wilmington, DE, USA) and mouse TNF-α Quantikine ELISA Kit (cat. no. SMTA00B; R&D Systems), respectively.

### Detection of *Vnn1* promoter methylation

Total DNA was extracted from lung tissues using the PureLink Genomic DNA Mini Kit (cat. K182001; Invitrogen/Thermo Fisher Scientific). 200 ng of DNA from each sample was bisulphite modified using the EZ DNA Methylation Kit (cat. no. D5001; Zymo Research, Irvine, CA, USA). The PCR reaction was performed using the specific primer pairs designed to amplify the target region (forward: tgttgtgattttgtttaaggata, reverse: tctaactataaaacaaaacaccttaac), 95°C/4 min; 40 cycles of 95°C/30 s, 85°C/30 s and 72°C/30 s; 72°C/5 min. The PCR product was purified using GenElute™ Gel Extraction Kit (cat. NA1111; Millipore/Sigma), and then cloned into pTG19-T vector for sequencing. The DNA methylation percentage was determined and compared.

### Reverse transcription-quantitative polymerase chain reaction (RT-qPCR)

Total RNA was extracted by using TRIzol Reagent (cat. no. 15596026; Invitrogen/Thermo Fisher Scientific). The concentration of RNA was determined by using the ND-1000 spectrophotometer (NanoDrop Technologies, Thermo Fisher Scientific). Totally, 2 μg RNA was reverse transcribed into cDNA by using the First-Strand cDNA Synthesis kit (cat. no. 11483188001; Sigma-Aldrich, St Louis, MO, USA). qPCR was performed using the SYBR Green PCR Master Mix (cat. no. 1725270; Bio-Rad Laboratories, Hercules, CA, USA) on the PE5700 Real-Time PCR system (Bio-Rad Laboratories). The specific primer pairs: *Vnn1* (forward 5′-ttatgcctttggagcctttg-3′, reverse 5′-agggaagacataccgggttc-3′, 184 bp); *β-actin* (forward 5′-ctgtccctgtatgcctctg-3′, reverse 5′-atgtcacgcacgatttcc-3′, 218 bp). The PCR program's initial denaturation step was conducted at 95°C for 10 min followed by 35 cycles of 95°C for 45 s, 58°C for 45 s and 72°C for 1 min. The mRNA expression levels of *Vnn1* were normalized with the housekeeping gene β-actin. The relative expression levels of the target genes were calculated using the 2^−ΔΔCt^ method.

### Chromatin immunoprecipitation (ChIP) assay

Chromatin was prepared from primarily cultured lung bronchial epithelial cells, and ChIP assay was performed according to the Abcam X-ChIP protocol. Briefly, cells were fixed with 1% formaldehyde for 10 min and neutralized with 1× glycine. Chromatin lysates were prepared, pre-cleared with Protein-A/G Sepharose beads, and immunoprecipitated with the antibodies against HNF-4α, PGC-1α, or normal rabbit IgG in the presence of bovine serum albumin (BSA) and salmon sperm DNA. The beads were extensively washed three times before reverse crosslinking. The immunoprecipitated DNA was purified using a PCR purification kit (cat. no. K310001; Invitrogen/Thermo Fisher Scientific) and subsequently quantified by real time PCR with the primers (forward: 5′-gctcaagcgaccctcctg-3′, reverse: 5′-catgctgaagtccaaaga-3′) flanking binding sites for HNF4α on the mouse *Vnn1* promoter.

### Immunoblot assay

Total proteins were extracted by using radioimmunoprecipitation assay buffer [25 mmol l^−1^ Tris-HCl pH 7.4, 150 mmol l^−1^ NaCl, 1% Nonidet-40, 0.1% SDS, 0.5% sodium deoxycholate (cat. no. D6750; Sigma-Aldrich)] supplemented with 1 mmol l^−1^ Na_3_VO_4_ (cat. no. S6508; Sigma-Aldrich), 1 g l^−1^ leupeptin (cat. no. L2884; Sigma-Aldrich) and 1 mmol l^−1^ PMSF (cat. no. 93842; Sigma-Aldrich). Nuclear proteins were prepared by using the CelLytic NuCLEAR Extraction kit (cat. no. NXTRACT, Sigma-Aldrich). Protein concentration was quantified by using the Pierce™ BCA Protein Assay Kit (cat. no. 23225; Thermo Fisher Scientific). A total of 75 μg protein was separated by using 12.5% or 7.5% SDS-PAGE, and then transferred to the Nitrocellulose Transfer Membrane (cat. no. ab133412; Abcam). The membranes were blocked at room temperature for 1 h in 5% low-fat milk or BSA prepared in tris-buffered saline containing 0.1% Tween-20 (TBST), and then incubated for overnight at 4°C with the following primary antibodies: rabbit anti-vannin-1 antibody (1:1000, cat. no. ab205912; Abcam), rabbit anti-PI3 Kinase p85 antibody (1:1000; cat. no. 4292; Cell Signaling Technology, Danvers, MA, USA), rabbit anti-phospho-PI3 Kinase p85 (Tyr458)/p55 (Tyr199) antibody (1:1000; cat. no. 4228; Cell Signaling Technology), rabbit anti-phospho-Akt^Ser473^ monoclonal antibody (1:500; cat. no. 4058; Cell Signaling Technology), rabbit anti-Akt polyclonal antibody (1:1000; cat. no. 9272s; Cell Signaling Technology), rabbit anti-PTEN polyclonal antibody (1:500; cat. no. 9552; Cell Signaling Technology), mouse anti-phospho-PTEN^Tyr366^ (1:500, cat.no. ab109454; Abcam), rabbit anti-IKB alpha antibody (1:500; cat. no. ab32518; Abcam), rabbit anti-PGC1α antibody ChIP grade (1:500, cat. no. NBP1-04676; Novus Biologicals, Centennial, CO, USA), mouse anti-HNF4-alpha antibody ChIP Grade (1:500, cat. no. ab41898; Abcam), rabbit anti-NFκB p65 subunit antibody (1:1000; cat. ab16502; Abcam), rabbit anti-histone H3 monoclonal antibody (1:1000; cat. no. 9717; Cell Signaling Technology), rabbit anti-GAPDH monoclonal antibody (1:10,000; cat. no. 5174S; Cell Signaling Technology) and mouse anti-β-actin antibody (1:5000; cat. ab8226; Abcam). Histone H3, β-actin, and GAPDH was used as the internal reference, respectively. Following three washes with TBST, the membranes were incubated with horseradish peroxidase (HRP)-conjugated goat anti-rabbit IgG (1:10,000; cat. no. G-21234; Invitrogen/Thermo Fisher Scientific) for 1 h at room temperature. Following three washes with TBST, the blots were developed by using the Enhanced Chemiluminescence Western Blotting Substrate (cat. no. 32109; Pierce, Thermo Fisher Scientific), and the intensity of the bands was quantified by using the ImageJ software (version 1.51s; National Institute of Health, Bethesda, MD, USA).

### Immunoprecipitation assay

Immunoprecipitation was performed for evaluation of HNF4α and PGC1α interactions. In total, 250 µg of nuclear protein was incubated under gentle rotation for overnight at 4°C with 2 µg of mouse anti-HNF4α antibody (2 µg of normal mouse IgG as the control), followed by 1 h incubation with 25 µl of Protein G-coupled agarose beads (cat. P3296; Millipore/Sigma). After five washes with 0.1% NP40/PBS, the beads were eluted with 25 µl of 2× SDS Loading buffer. The elution was stored at −80°C for further immunoblot analysis.

### DCF assay

The levels of reactive oxygen species (ROS) was quantitatively measured using The OxiSelect™ *In Vitro* ROS/RNS Assay Kit (cat. no. STA-347-5; CELL BIOLABS, San Diego, CA, USA) according to the manufacturer's guidelines. In this study, 2×10^7^ cells/ml and 50 mg/ml tissue lysates were prepared, and 50 µl was assayed in duplicate. Read the relative fluorescence with SpectraMax Gemini XS Fluorometer (Molecular Devices, San Jose, CA, USA) at 480 nm excitation/530 nm emission. Data are presented as the fold change over the controls.

### Knockdown of Vnn1 in primary cultured lung cells

Tracheobronchial epithelial cells were isolated from the IUGR mice injected with OVA or PBS according to previously described protocol (Horani et al., 2013; Lam et al., 2011). The primary tracheobronchial epithelial cells were cultured in DMEM/F12 media (cat. no. 51445C; Sigma-Aldrich) supplemented with 10% FBS, 15 mM HEPES, 4 mM glutamine, 0.03% NaHCO3, 1× penicillin/streptomycin, and 1× Fungizone solution. The validated *VNN1* MISSION shRNA Lentiviral Transduction Particles (cat. SHCLNV-NM_011704, TRCN0000094464, 10^6^TU; Sigma-Aldrich) were used for knockdown of *Vnn1*, and the MISSION TRC2 pLKO.5-puro Empty Vector Control Transduction Particles (cat. SHC201v; Sigma-Aldrich) were used as the controls. After 48 h transfection, cells were harvested for evaluation.

### Statistical analysis

Results are presented as the mean±s.d. The unpaired *t*-test was used for comparison between two groups. One-way analysis of variance (ANOVA) followed by the Kruskal–Wallis test and two-way ANOVA with the Sidak correction were used for multiple comparisons (Prism 6.0, GraphPad, USA). The value *P*<0.05 was considered as statistically different.
